# Low Frequency of Fruit and Vegetable Consumption Among Canadian Youth: Findings From the 2012/2013 Youth Smoking Survey

**DOI:** 10.1111/josh.12359

**Published:** 2016-01-13

**Authors:** Leia Minaker, David Hammond

**Affiliations:** a Propel Centre for Population Health Impact, University of WaterlooWaterloo, Ontario, Canada; b School of Public Health and Health Systems, University of WaterlooWaterloo, Ontario, Canada

**Keywords:** diet, adolescents, surveillance, Canada, fruit and vegetable consumption

## Abstract

**BACKGROUND:**

Frequent fruit and vegetable (FV) consumption is protective against some cancers, cardiovascular disease, and other chronic diseases. This study explores self-reported frequency of FV consumption in a nationally generalizable sample of Canadian youth in grades 6-12.

**METHODS:**

Data from grades 6-12 students who participated in the 2012-2013 Youth Smoking Survey (N = 47,203) were used to examine frequency of FV consumption. Logistic regression models were fitted to examine differences in meeting national FV intake recommendations by sociodemographic, student, and regional characteristics.

**RESULTS:**

Approximately 10% of Canadian grade 6-12 students met FV recommendations. Students in grades 6 and 7 had significantly higher odds of meeting recommendations relative to students in grades 8-12. Students who reported achieving “mostly As” on their report cards had significantly higher odds of meeting FV recommendations relative to those receiving As and Bs, Bs and Cs, or Cs (OR = 0.71, OR = 0.53, and OR = 0.46, respectively, p < .0001 for each). Students in British Columbia and Ontario had higher odds of meeting recommendations relative to students in Newfoundland, Prince Edward Island, and Nova Scotia.

**CONCLUSIONS:**

Only 1 in 10 Canadian youth are meeting FV recommendations. Programs and policies to encourage FV consumption are required to help mitigate future health issues associated with inadequate FV consumption.

Fruit and vegetable (FV) consumption is associated with reduced risk for chronic diseases including some cancers, cardiovascular disease, obesity, and all-cause mortality.^1^–^5^ Worldwide, low fruit consumption ranked fifth in terms of causes of disability-adjusted life years in the 2010 Global Burden of Disease study, accounting for 4.9 million deaths and 4% of global disability-adjusted life years.^6^ In Canada, as in other countries,^7^ residents generally fail to meet recommended daily guidelines for fruit and vegetables.^8^–^12^ Canada's Food Guide recommends a diet rich in fruits and vegetables to reduce the risk of chronic disease^13^ and provides age- and sex-specific recommendations for FV consumption, ranging from 4 servings per day for boys and girls aged 2-3 years to 8-10 servings per day for men aged 19-50 years. An estimated 22,000 deaths in Canada could be averted or delayed by adhering to guidelines on FV consumption proposed by Health Canada.^12^

FV consumption is often used as an indicator of healthy diet consumption among children and youth because collecting comprehensive dietary data is time- and resource-intensive.^14^–^16^ In terms of nationally representative data on adolescents, data from the Canadian Community Health Survey (CCHS) showed that in 2004 (the latest year 24-hour diet recall data were collected in Canada), the majority of children and youth in Canada did not consume the minimum number of FV servings recommended based on age and sex.^9^ Food frequency data from the 2012 CCHS showed that among 12- to 19-year-olds, 43.5% of boys and 46.2% of girls reported consuming fruits and vegetables at least 5 times per day.^17^ Other nationally representative data taken from the Youth Smoking Survey 2010/2011 found that 94% of Canadian grades 9-12 students did not meet the age- and sex-specific food guide recommendations for FV consumption.^18^ These metrics are not altogether comparable, because youth 12 and older are recommended to eat at least 6 servings of fruits and vegetables every day.^13^ The Food Guide recommends 6 servings per day for youth aged 9-13, 7 servings for girls aged 14-18, and 8 servings for boys aged 14-18.^13^

A large body of literature has identified schools as ideal environments in which to promote consumption of fruits and vegetables.^19^–^22^ Qualitative research from several countries consistently suggests that schools fail to adequately promote fruit and vegetables, and FV availability and quality in schools are poor.^23^ Currently in Canada, developing strategies to increase FV consumption among school-aged youth is a priority.^24^ Promising strategies have been identified, including school FV snack programs.

The objective of the current paper was to examine FV consumption and predictors of meeting FV recommendations in a 2012-2013 nationally generalizable, school-based sample of Canadian grades 6-12 students.

## METHODS

### Procedure

The 2012-2013 Youth Smoking Survey (YSS) is a nationally generalizable school-based, paper-and-pencil survey that is used to measure the determinants of smoking and other health risk behaviors among youth.^25^,^26^ The target population was students in grades 6 through 12 (ages 11-17) at all schools (including public, Catholic, and private schools) (N = 485) in 9 provinces. Those residing in Manitoba, Yukon, Nunavut, and Northwest Territories and those living in institutions or on First Nations reserves were excluded in 2012-2013. Surveys were pilot tested to assess the logic and student understanding of the questions. Approximately 73% of respondents participated with passive parental permission, and 27% participated with active parental permission. The majority of schools required only passive permission (N = 259), 156 schools required only active permission, and 35 schools required some students to participate via active permission and other students to participate via passive permission. The response rate (% of completed surveys out of all eligible students) for students participating with active permission was 49%. The response rate for students participating with passive permission was 82%. The YSS survey was administered during class time, and participants were not remunerated. Survey development, design, weights, response rates, and data collection protocol for the 2008 YSS have been published.^25^ On average, 64% of schools that were approached participated (range, 38% in Ontario to 96% in Newfoundland), and 73% of eligible students completed questionnaires. The 2012-2013 YSS was administered to 47,203 youths in grades 6 through 12 attending schools from 9 (of 10) Canadian provinces (in Quebec, secondary school ends at grade 11). Data were collected between October 2012 and June 2013. Data were analyzed in 2014.

### Instrumentation and Participants

We assessed FV consumption by using the YSS survey question: “On an usual day, how many servings of fruits and/or vegetables do you eat? (Include fresh, frozen, canned, and cooked items like apple, banana, carrot, salads, and 100% juice. Do not include chips, French fries, or other fried potatoes).” Response options included: 0 servings; 1-2 servings; 3-4 servings; 5 servings; 6 servings; 7 servings; 8 or more servings, and a highlighted text box of examples of single servings specified “1/2 cup of fresh, frozen, or cooked vegetables,” “1 cup of raw leafy vegetables; like a small salad,” “1 medium fruit; like an apple, pear or banana,” and “1/2 cup of 100% fruit or vegetable juice.” A dichotomous outcome was created according to Canada's Food Guide to Healthy Eating (CFG) age- and sex-specific FV intake recommendations. The CFG recommends 6 servings of FV per day for children aged 9-13, 7 FV servings for girls aged 14-18, and 8 FV servings for boys aged 14-18.^13^ Data were derived as 1: student usually meets sex- and age-specific FV intake recommendations and 0: student does not usually meet sex- and age-specific recommendations.

Sociodemographic and regional correlates included the respondent's sex, grade (6-12), province of residence, self-reported race/ethnicity (white, black, Asian, Aboriginal, Latin American, or “other”), self-reported academic achievement, and the amount of weekly spending money received. Students chose one of the following options to identify their academic achievement: Mostly As, As and Bs, Bs and Cs, Cs, and Ds. Although grade categories were overlapping, which is not ideal, students only chose one option and pilot testing of the survey instrument revealed a high level of understanding of the question. Weekly spending money was categorized as none, $1 to $10, $11 to $20, $21 to $40, and more than $40 (in Canadian currency).

### Data Analysis

Survey weights were used to adjust for sample selection (school and class levels), nonresponse (school, class, and student levels), and post-stratification of the sample population relative to grade and sex distribution in the total population. Descriptive statistics were used to show the percent of Canadian students meeting FV intake recommendations as well as the percent of students reporting each unique response option by sex, grade, province of residence, self-reported race, self-reported academic achievement, and weekly spending money. Finally, a logistic regression model was fitted to examine independent variables related to the odds of meeting FV intake recommendations. Correlates included sex, grade, province of residence, self-reported race, self-reported academic achievement, and weekly spending money. The model examined students' meeting FV intake recommendations among all students.

Assumptions of logistic regressions (eg, sufficient sample size for single cell counts) were checked, and goodness-of-fit tests were used to check model fit. Logistic regressions were conducted by using PROC SURVEYLOGISTIC in SAS 9.3.2 (SAS Institute Inc, Cary, NC). Because of the large sample size, statistical significance was defined as p < .01.

## RESULTS

### Sample

Table1 shows the sociodemographic and reported FV intake of the 47,203 students in the current study. Overall, 9.9% of Canadian students in grades 6-12 reported meeting CFG FV intake recommendations. The majority of students (65.5%) reported usually consuming 1-4 FV servings per day.

**Table 1:** Sample Descriptive Statistics, Grades 6-12, Canada, 2012-2013 Youth Smoking Survey

Characteristics of survey population	N (unweighted) (% weighted)	Meet FV recommendations (weighted %)	0 servings (weighted %)	1-2 servings (weighted %)	3-4 servings (weighted %)	5 servings (weighted %)	6 servings (weighted %)	7 servings (weighted %)	8+ servings (weighted %)
Canada	47,203 (100)	9.9	3.1	27.8	37.7	15.8	7.5	3.3	4.8
Sex									
Girls	23,996 (48.7)	10.7	2.0	27.7	38.9	15.9	7.9	3.4	4.2
Boys	23,207 (51.3)	9.2	4.2	27.8	36.6	15.7	7.1	3.2	5.4
Grade									
6	6146 (12.7)	17.9	1.7	25.0	38.3	17.0	7.7	3.9	6.3
7	6816 (14.0)	17.1	2.6	25.9	38.0	16.2	8.6	3.6	5.0
8	6837 (14.5)	10.9	2.8	27.8	39.9	16.3	6.8	2.6	3.8
9	7066 (15.1)	5.6	4.2	28.7	37.3	15.1	7.4	2.9	4.4
10	7680 (15.0)	6.3	2.6	28.1	38.2	15.7	7.2	3.8	4.4
11	7114 (15.0)	6.6	3.3	27.9	37.6	15.0	7.4	3.6	5.2
12	5544 (13.8)	6.8	4.3	30.7	34.6	15.4	7.1	2.8	5.0
Provinces									
Newfoundland	4265 (1.4)	8.7	4.9	32.6	35.2	14.3	6.2	2.4	4.3
Prince Edward Island	2525 (0.5)	7.4	3.3	30.8	39.2	14.6	6.1	2.7	3.3
Nova Scotia	4600 (2.7)	8.3	3.3	31.7	37.8	13.2	7.4	2.4	4.3
New Brunswick	3716 (2.6)	9.4	4.9	30.8	36.7	13.0	7.1	2.6	5.0
Quebec	6158 (19.7)	9.6	3.7	26.7	36.6	17.7	7.6	3.5	4.1
Ontario	8048 (45.4)	10.2	3.0	27.9	37.5	15.6	7.5	3.4	5.1
Saskatchewan	5638 (3.2)	9.4	3.1	29.2	37.9	14.8	7.2	3.1	4.6
Alberta	5743 (11.4)	9.9	2.0	27.0	40.0	15.3	7.5	3.2	5.0
British Columbia	6510 (13.1)	10.3	3.0	27.3	38.3	15.8	7.2	3.3	5.1
Ethnicity									
White	33,557 (64.7)	9.5	2.6	27.5	37.8	16.7	7.7	3.3	4.5
Black	1971 (6.7)	10.4	7.2	30.8	34.1	11.4	6.7	4.3	5.6
Asian	4621 (12.1)	11.6	2.6	27.2	39.6	14.9	7.6	3.3	4.8
Aboriginal	3072 (4.7)	10.2	5.1	29.9	37.0	12.0	6.8	3.2	6.0
Latin American	708 (2.5)	13.9	3.7	23.2	38.9	13.9	8.8	4.0	7.5
Other	2893 (8.3)	11.6	4.7	26.6	35.3	16.9	7.0	3.2	6.4
Academic achievement									
Mostly As	14,332 (26.2)	13.0	2.0	19.7	37.9	20.0	10.1	4.5	5.8
Mostly As/Bs	20,365 (46.9)	9.7	1.9	28.3	39.6	15.2	7.2	3.2	4.6
Mostly Bs/Cs	8328 (18.4)	7.0	4.5	35.9	35.3	13.1	5.0	2.1	4.0
Mostly Cs	1899 (3.4)	6.2	5.5	38.7	32.1	11.7	5.2	2.2	4.4
Mostly below Cs	630 (1.2)	11.4	29.9	25.5	21.8	8.5	5.5	1.1	7.9
Weekly spending money									
No money	8931 (21.0)	9.2	3.7	30.5	38.3	14.4	6.3	2.8	4.1
$1 to $10	8657 (18.5)	10.1	2.1	28.3	39.7	16.4	7.0	2.9	3.4
$11 to $20	6682 (13.1)	10.3	2.2	26.4	37.8	17.6	8.1	3.7	4.4
$21 to $40	5284 (10.2)	9.9	2.0	25.4	38.2	17.3	8.4	3.6	5.1
More than $40	8581 (17.6)	10.2	3.5	25.2	35.5	15.7	8.8	4.0	7.2

FV, fruit and vegetable.

### Meeting FV Intake Recommendations

There were no significant differences between girls and boys in terms of meeting FV intake recommendations (Table2). Grade 6 students had significantly higher odds of meeting FV intake recommendations relative to grades 8-12 students. There were several provincial differences in the percent of students meeting FV intake recommendations. For example, relative to students in Ontario, students in 3 of the East Coast provinces (NL, PEI, NS) had significantly lower odds of meeting recommendations (OR = 0.69 p = .0001, OR = 0.70 p = .0005, OR = 0.72 p = .0003, respectively). Relative to students receiving no spending money per week, students receiving $11 to $20, $21 to $40, and more than $40 per week all had significantly higher odds of meeting recommendations (OR = 1.40, p = .0002, OR = 1.60 p < .0001, OR = 2.15 p < .0001, respectively). Finally, students who reported achieving “mostly As” on their report cards had significantly higher odds of meeting FV intake recommendations relative to those receiving As and Bs, Bs and Cs, or Cs (OR = 0.71, OR = 0.53, and OR = 0.46, respectively, p < .0001 for each). There were few significant differences by self-reported race. Students identifying as “other” had significantly higher odds of meeting recommendations compared with students identifying as White (OR = 1.33, p = .0098). Similarly, students identifying as Aboriginal and Latin American had significantly higher odds of meeting FV intake recommendations compared to students identifying as White (OR = 1.25, p = .042 and OR = 1.56, p = .0164, respectively).

**Table 2:** Logistic Regression Analysis of Variables Related to the Odds of Meeting Fruit and Vegetable Consumption Recommendations. Grades 6-12, Canada, 2012-2013 YSS

Predictors	Meet FV recommendations (%)	Meeting FV recommendations among all students (Model 1: N = 36,455)OR adjusted (99% CI)	p Value
Sex			
Girls (ref)	10.7	1.0	
Boys	9.2	0.89 (0.77, 1.03)	.0450
Grade			
6 (ref)	17.9	1.0	
7	17.1	0.92 (0.75, 1.14)	.3188
8	10.9	0.51 (0.41, 0.65)	< .0001
9	5.6	0.23 (0.18, 0.31)	< .0001
10	6.3	0.25 (0.18, 0.33)	< .0001
11	6.6	0.22 (0.16, 0.30)	< .0001
12	6.8	0.22 (0.15, 0.31)	< .0001
Provinces			
Ontario (ref)	8.7	1.0	
Newfoundland	7.4	0.69 (0.54, 0.89)	.0001
Prince Edward Island	8.3	0.70 (0.53, 0.91)	.0005
Nova Scotia	9.4	0.72 (0.57, 0.91)	.0003
New Brunswick	9.6	0.89 (0.71, 1.12)	.2005
Quebec	10.2	0.83 (0.68, 1.02)	.0186
Saskatchewan	9.4	0.83 (0.66, 1.05)	.0416
Alberta	9.9	0.91 (0.73, 1.13)	.2544
British Columbia	10.3	0.99 (0.81, 1.21)	.9171
Ethnicity			
White (ref)	9.5	1.0	
Black	10.4	1.18 (0.85, 1.64)	.1850
Asian	11.6	1.11 (0.88, 1.42)	.2464
Aboriginal	10.2	1.25 (0.95, 1.65)	.0402
Latin American	13.9	1.56 (0.97, 2.52)	.0164
Other	11.6	1.33 (1.01, 1.78)	.0098
Weekly spending money			
No money (ref)	9.2	1.0	
$1 to $10	10.1	1.03 (0.84, 1.26)	.7515
$11 to $20	10.3	1.40 (1.11, 1.77)	.0002
$21 to $40	9.9	1.60 (1.24, 2.06)	< .0001
More than $40	10.2	2.15 (1.68, 2.75)	< .0001
Self-reported grades			
Mostly As (ref)	13.0	1.0	
As and Bs	9.7	0.71 (0.59, 0.84)	< .0001
Bs and Cs	7.0	0.53 (0.42, 0.66)	< .0001
Cs	6.2	0.46 (0.28, 0.74)	< .0001
Cs and Ds	11.4	0.97 (0.52, 1.82)	.9035

CI, confidence interval; FV, fruit and vegetable; N, number; OR, odds ratio; YSS, Youth Smoking Survey.

Pairwise comparisons revealed that the odds of meeting FV recommendations were greater in the following groups: grades 6 and 7 (vs grades 8-12), grade 8 (vs grades 9-12), and students receiving mostly As and Bs (vs Bs and Cs). All pairwise comparisons between students in different money per week categories were significant at p < .01, except for 2 comparisons: $0 vs. $1 to $10, and $11 to $20 vs. $21 to $40. Pairwise comparisons of provinces revealed that students in British Columbia had significantly higher odds of meeting FV intake recommendations relative to those in Newfoundland, Prince Edward Island, and Nova Scotia.

## DISCUSSION

One in 10 Canadian students in grades 6-12 met Food Guide FV consumption recommendations in 2012-2013, which is concerning given the detrimental public health impacts associated with inadequate FV consumption.^12^ In the United States in 2010, only 17% of high school students consumed fruit at least 4 times daily and only 11% consumed vegetables at least 4 times daily.^27^ In 2003-2004, only 6% of American adolescents met either fruit or vegetable recommendations based on 24-hour diet recall data.^28^ Reasons for inadequate FV consumption range from parental intake and home accessibility^29^ to youths' access to unhealthy foods to the symbolic value of food for youths' image.^30^ Improving FV consumption among youth will clearly require concerted, multi-sector, multisetting efforts to provide opportunities for consumption.^31^ Using a nationally generalizable dataset, we found odds of meeting recommendations varied by grade, by weekly spending money, by self-reported race, and by self-reported academic achievement. Each of these findings is described in more detail below.

Students in lower grades had significantly higher odds of meeting recommendations compared with students in higher grades. Whereas at least 17% of grades 6 and 7 students were meeting recommendations, less than 7% of grades 9-12 students met recommendations. These findings are consistent with previous prospective research finding that FV consumption actually decreases over time,^11^,^32^ and have implications for school nutrition policies and programs. The school environment contributes to dietary outcomes in children,^19^,^33^,^34^ with lunch programs and selected state and local nutrition policies increasing FV consumption.^33^ In Canada, although many provinces have nutrition policies related to foods sold at schools (eg, British Columbia's Guidelines for food and beverage sales in schools or Ontario's Nutrition Standards for Ontario Schools^35^,^36^), the same nutrition criteria do not apply to foods provided at school through free school breakfast or lunch programs. Moreover, implementation of school nutrition policies is inconsistent at best, with many schools failing to meet policy requirements.^37^ Concerted efforts should be undertaken to establish, implement, and evaluate coherent nutrition policies for foods sold and provided at schools to improve FV consumption.^38^

Because the YSS does not collect data on family socioeconomic status, we were unable to examine socioeconomic status and FV consumption in the current study. However, FV consumption varied significantly by weekly spending money, with students who reported more money per week more likely to meet FV recommendations. It is possible that weekly spending money is a proxy for family income, because family income is associated with increased FV consumption.^29^ However, data from Scotland suggested that weekly spending money may be associated with fruit consumption differently than other measures of socioeconomic position.^39^ Specifically, weekly spending money was unrelated to fruit consumption, whereas measures of family affluence were indeed positively associated with fruit consumption.^39^ Our finding that weekly spending money was associated with students' odds of meeting FV recommendations does not support Currie et al's findings, perhaps due to different study contexts (Scotland vs Canada). Alternatively, the difference may be explained by the different age ranges of students (11 to 15 years in Currie et al's study vs 11 to 17 years in ours). Age range notwithstanding, this finding warrants that attention continue to be paid to the impact of low socioeconomic position on FV intake.^40^,^41^ To create equitable school-based programs to address socioeconomic position and FV intake, schools face the challenge of creating policies and programs that support nutrition among economically disadvantaged children while avoiding stigma experienced by children receiving free school meals.^42^

Third, students self-identifying as Aboriginal, Latin American, and Other had significantly higher odds of meeting FV recommendations compared with white students. In one study from Montreal, Canada, family origin was significantly associated with children's FV consumption, although results were not consistent between immigrant groups.^43^ In a national study of Canadian adolescents, data from 2003 indicated that cultural/racial origin was not significantly associated with FV intake.^44^ Our finding that FV intake was highest among youth identifying as Latin American is consistent with a substantial body of research showing that FV intake among low-income youth is highest among Hispanic youth compared with white and African-American youth,^45^ although other reviews have found that ethnicity shows inconsistent associations with youths' FV intake.^29^

Finally, and perhaps one of the most interesting findings of the current study, students who reported high academic achievement had significantly higher odds of meeting FV recommendations after controlling for sex, grade, province of residence, and weekly spending money. Students who reported receiving mostly Cs had half the odds of students reporting mostly As of meeting FV recommendations. Among children and youth, academic performance has been positively associated with adherence to the Mediterranean diet,^46^ overall diet quality,^47^ and FV consumption.^48^,^49^ Participation in school breakfast programs has been associated with higher academic achievement in the only study to our knowledge that has evaluated the impact of a school food provision program on academic performance.^50^,^51^ If school meal provision consumption indeed plays a role in improving academic performance, the current health-related framing of FV provision could be broadened to include the education sector in addition to the health sector. Despite differences in consumption by grade, weekly spending money, and academic achievement, the overall picture is grim, with about 90% of Canadian grades 6-12 students failing to meet recommendations.

This study has several limitations common to survey research. First, cross-sectional survey data like the YSS data described here do not allow causal inferences to be made. Second, in schools where active permission was required for student participation, response rates were relatively low (50%). Problems associated with active permission is a common issue in school-based research. Researchers should continue to argue for passive permission procedures in future school surveys. Third, as noted, participants' family socioeconomic status was not captured in the YSS, which is a limitation given that dietary outcomes vary by socioeconomic status.^19^,^29^ Fourth, the FV intake outcome measure has not been previously validated, although honest reporting was encouraged through strict confidentiality procedures throughout data collection. In addition, the question is consistent with surveillance measures employed by Health Canada and the Public Health Agency of Canada, which assess frequency of FV consumption rather than amounts. Strengths of this study include a large sample with provincially generalizable estimates of FV consumption.

Future strategies to increase FV consumption should include input from multiple stakeholders, including national and provincial governments, as well as nongovernmental health advocacy organizations and stakeholders from the produce industry. School food policies and programs should be evidence-based with a focus on equity to decrease disparities in healthy diets in Canada and to decrease future morbidity and mortality. Additionally, future research should use validated measures of dietary data collection, such as the ASA24, an online 24-diet recall^52^,^53^ to ensure high-quality, continued surveillance on dietary behaviors of Canadian youth.

## IMPLICATIONS FOR SCHOOL HEALTH

Only 1 in 10 Canadian youth are consuming fruits and vegetables frequently enough to meet national recommendations. Youth who meet recommendations are more likely to be younger and to get good grades relative to those who do not meet recommendations. School-based programs to increase FV consumption are needed, especially among older students and students living in the Atlantic provinces. Schools should ensure that school food programs include fruits and vegetables. Schools should explore partnerships with food-related nongovernmental organizations and FV industries to improve accessibility of fruits and vegetables within schools.

### Human Subjects Approval Statement

The University of Waterloo Human Research Ethics Committee, the Health Canada Research Ethics Board, and appropriate school board and public health ethics committees approved this study.
